# Identification of prognosis‐related alternative splicing events in kidney renal clear cell carcinoma

**DOI:** 10.1111/jcmm.14651

**Published:** 2019-09-05

**Authors:** Yongdi Zuo, Liang Zhang, Weihao Tang, Wanxin Tang

**Affiliations:** ^1^ Department of Nephrology West China Hospital Sichuan University Chengdu China; ^2^ Chengdu Foreign Language School Chengdu China

**Keywords:** alternative splicing, bioinformatics, KIRC, LASSO regression, TCGA

## Abstract

Alternative splicing (AS) contributes to protein diversity by modifying most gene transcriptions. Cancer generation and progression are associated with specific splicing events. However, AS signature in kidney renal clear cell carcinoma (KIRC) remains unknown. In this study, genome‐wide AS profiles were generated in 537 patients with KIRC in the cancer genome atlas. With a total of 42 522 mRNA AS events in 10 600 genes acquired, 8164 AS events were significantly associated with the survival of patients with KIRC. Logistic regression analysis of the least absolute shrinkage and selection operator was conducted to identify an optimized multivariate prognostic predicting mode containing four predictors. In this model, the receptor‐operator characteristic curves of the training set were built, and the areas under the curves (AUCs) at different times were >0.88, thus indicating a stable and powerful ability in distinguishing patients' outcome. Similarly, the AUCs of the test set at different times were >0.73, verifying the results of the training set. Correlation and gene ontology analyses revealed some potential functions of prognostic AS events. This study provided an optimized survival‐predicting model and promising data resources for future in‐depth studies on AS mechanisms in KIRC.

## 1 INTRODUCTION

Alternative splicing (AS) modifies over 90% of human genes by removing most introns and selectively including or excluding specific exons.^1^, ^2^, ^3^ It regulates the specificity of gene expression,^4^ plays an important role in the diversity of mRNA isoforms with a limited set of genes and down‐regulates the translation of mRNA isoforms.^5^, ^6^, ^7^ AS is also an essential mechanism in physiological processes, such as hematopoiesis and muscle function,^8^, ^9^ and in cancer‐causing pathological processes, including proliferation, apoptosis, hypoxia, angiogenesis, immune escape and metastasis.^10^, ^11^ Furthermore, changes and defects in splicing patterns and generation of peculiar mRNA isoforms can trigger cancer.^11^, ^12^, ^13^ Thus, AS participates in oncogenesis, and the profiling of AS events may provide novel potential biomarkers for cancer prognosis, diagnosis and treatment.

In 2012, the estimated number of new cases of kidney cancer worldwide was 338 000.^14^ Currently, kidney cancer is the 9th most common carcinoma in men and the 14th most common in women globally. Renal cell carcinoma comprises more than 90% of this malignant tumour, and clear cell carcinoma accounts for a large proportion of approximately 70%.^15^ The relevance of AS events in kidney cancer is gradually being revealed. The importance of the AS of enhancer of zeste 2 polycomb pre‐mRNA in the tumorigenic potential of kidney cancer has been reported.^16^ The AS variants of doublecortin‐like kinase 1 are overexpressed in kidney renal clear cell carcinoma (KIRC) and may be implicated in immune response.^17^ In addition, differentially splicing isoforms between KIRC and non‐tumour tissues have been studied, and a cassette exon differentially skipped in DAB adaptor protein 2 in KIRC has been identified.^18^ The importance of the systemic profiling of survival‐associated AS has been emphasized in lung, ovarian and prostate cancers.^19^, ^20^, ^21^ However, the survival‐associated AS events in KIRC have yet to be systematically studied.

Bioinformatics analysis is a scientific method to examine genetic information and employ approaches to acquire, store, visualize and interpret medical or biological data.^22^ Bioinformatics is widely used in cancer studies.^23^, ^24^ Machine learning, which is an important bioinformatics method, is applied to considerable clinical data sets to develop robust risk models and redefine patient classes.^25^ The least absolute shrinkage and selection operator (LASSO) regression is one of the machine learning methods suitable for the regression of massive and multivariate variables.^26^ These methods provide an optimized approach for the systematic study of AS in KIRC.

The Cancer Genome Atlas (TCGA) is a project that provides the detailed mRNA expression data and clinical information of patients with cancer.^27^ In the current study, we comprehensively profiled the genome‐wide AS events in 537 patients with KIRC from TCGA. We discovered a number of survival‐associated AS events through bioinformatics analysis. Using LASSO regression, we built a high‐power prognostic model based on the per cent spliced in (PSI) value of AS for patients with KIRC. We also revealed interesting function pathways in correlative genes and the potential mechanisms of AS influencing the prognosis of such patients. We aimed to construct an optimized survival‐predicting model and provide useful data resources for future in‐depth studies on AS mechanisms in KIRC.

## 2 MATERIALS AND METHODS

### 2.1 Data acquisition and processing

First, the RNA‐seq data and clinical information of the KIRC cohort were downloaded from the TCGA data portal (https://tcga-data.nci.nih.gov/tcga/) by using the GDC tool. mRNA expression counts were converted into an expression value by using DEseq R package, and only the expression counts of more than 2 were included.^28^ Furthermore, only patients with at least 30 days of overall survival (OS) were retained in the study. Second, AS events with PSI value of KIRC were obtained from the TCGA SpliceSeq data portal (https://bioinformatics.mdanderson.org/TCGASpliceSeq/PSIdownload.jsp). AS events were divided into seven different types, so the intersections between these types and the quantitative analysis of these interactive sets were presented by the UpSet plot.^29^ PSI value is a visualized ratio for quantifying an AS event from 0 to 1, and it is calculated for the seven types of AS events. A PSI value of 0 or 1 was then filtered, and the corresponding splicing patterns whose exon was NA were deleted. Third, a matrix with the seven types of AS events was built. The PSI value of the AS events was combined with the OS information and survival stage by gene symbols.

### 2.2 Univariate survival analysis

COX regression allows us to calculate a special form of rate ratios known as hazard ratios (HRs) and investigate the relationship of predictors and time to event. Using COX regression, we can obtain a *P* value provided by the log‐rank test and estimate an effect with its confidence intervals (CIs). To determine the survival‐associated AS events, we performed univariate COX regression analysis between AS and OS by using ‘survival’ and ‘survminer’ R package. Furthermore, we divided the survival‐associated AS events into high‐ or low‐risk groups by using the surv_cutpoint function and applied a log‐rank test to verify the cut‐point accuracy. Kaplan‐Meier (K–M) curves were built to demonstrate the survival probability variation with time in high‐ and low‐risk groups. *P* < .05 was considered significant, and all reported *P* values were two‐sided.

### 2.3 LASSO regression for multivariate prognostic model construction

To gain the final highly important prognostic predictors with less errors, we applied LASSO regression rather than multivariate COX regression, which may not be that suitable for this type of data, among survival‐associated AS events.^30^ This process, which is one of the machine learning methods adopted in several studies, was performed using glmnet package in R.^31^, ^32^ A multivariate regression formula was constructed on the basis of the PSI value of AS events. Lastly, several notable predictors with non‐zero LASSO coefficients were obtained.

### 2.4 Training and testing for multivariate prognostic models

The tumour samples obtained from the TCGA data portal were randomly distributed into two parts, namely training and test groups, by using classification and regression training (caret) packages. In the training set, a risk score was calculated for each patient, and the data were divided into high‐ and low‐risk groups based on the cut‐point of a risk score. The K‐M curve was then applied to verify whether the prognostic model could distinguish patients with long or short OS.^33^ Receiver operator characteristic (ROC) curves in different years were built by using the survival ROC package to assess the efficiency of the prognostic model. In the test group, the same processes were performed to validate the prognostic model of this group.

### 2.5 Correlation analysis for the source genes of AS

After several final important prognostic AS predictors were obtained, the correlation between the expression levels of AS source genes and human protein‐code genes was analysed through Pearson correlation analysis. *P* < .05 was identified to be correlative genes.

### 2.6 Gene ontology (GO) analysis

Gene ontology (GO) analysis includes three categories: molecular function (MF), biological process (BP) and cellular component (CC). In this study, we selected BP to perform GO analysis through an enrich GO function in the clusterProfiler package(version 3.5) and obtained the original database from ‘org.Hs.eg.db’ package.^34^ To speculate the potential functions of survival‐associated AS events leading to KIRC, we chose the source genes of notable AS events whose univariate regression *P* < .01 to perform GO analysis. In addition, to speculate the potential functions of the final AS predictors, we selected the top 500 correlative genes to perform GO analysis. For the AS events in GO analysis, we considered the adjusted *P* < .05 significant.

### 2.7 Statistical analysis

R‐studio platform (v. 3.5.1) was used for UpSet plot, univariate Cox regression, LASSO regression, K‐M curves, ROC analysis, Pearson correlation and GO analysis. *P* < .05 indicated statistically significant difference. Regression coefficient (R) >0.5 was the cut‐off of correlation analysis.

## 3 RESULTS

### 3.1 Overview of AS events in KIRC cohort

**Figure 1:**
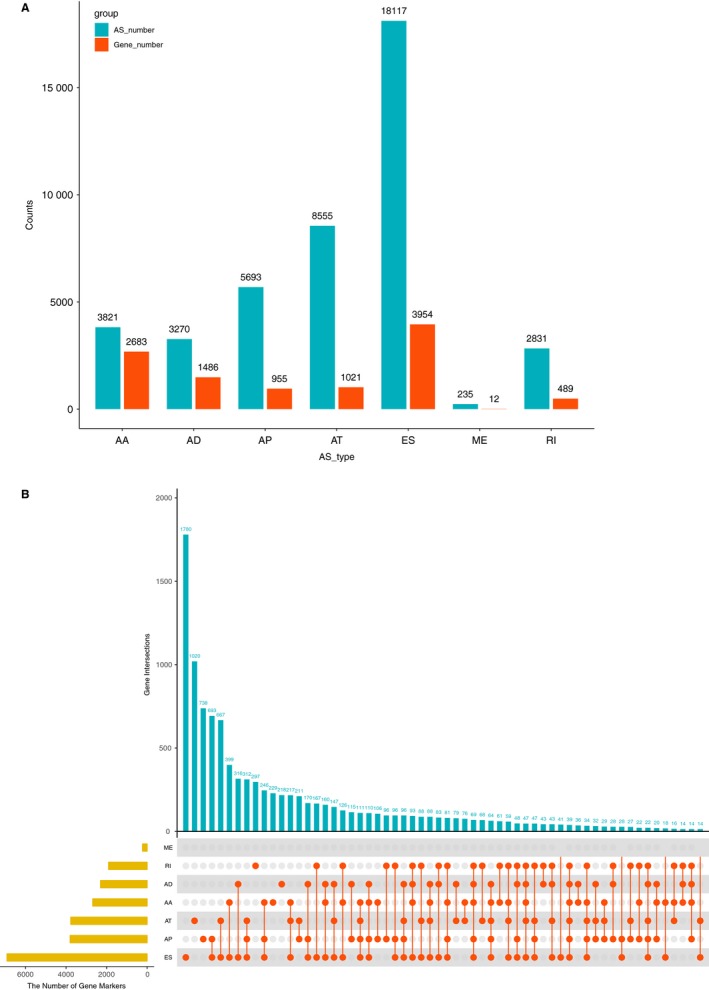
AS events in KIRC. A, AS events were divided into seven types. Blue columns represent AS number, and red columns represent gene number. B, Interactions between the seven types of AS events in KIRC presented by UpSet plot. Red dots represent splicing type, yellow columns represent AS number, and blue columns represent the interactive number of AS events

AS events were comprehensively analysed in 537 patients with KIRC. AS events were divided into seven types, namely alternative acceptor site (AA), alternative donor site (AD), alternative promoter (AP), alternative terminator (AT), exon skip (ES), mutually exclusive exons (ME) and retained intron (RI), as presented in (Figure 1A). In the KIRC cohort, we obtained 42 522 mRNA splicing events in 10 600 genes containing 3821 AAs in 2683 genes, 3270 ADs in 1486 genes, 5693 APs in 955 genes, 8555 ATs in 1021 genes, 18 117 ESs in 3954 genes, 235 MEs in 12 genes and 2831 RIs in 489 genes (Figure 1 A). Nearly half of the AS events was ES, demonstrating that ES was the prevalent type. In Figure 1B, most of the AS events were from one gene, which could have several types of AS events. For example, one gene might contain up to six AS types, such as ES, AP, AT, AA, AD and RI. Furthermore, ES was the most common type of AS, whereas ME was the least.

### 3.2 Survival‐associated AS events and GO analysis

**Figure 2:**
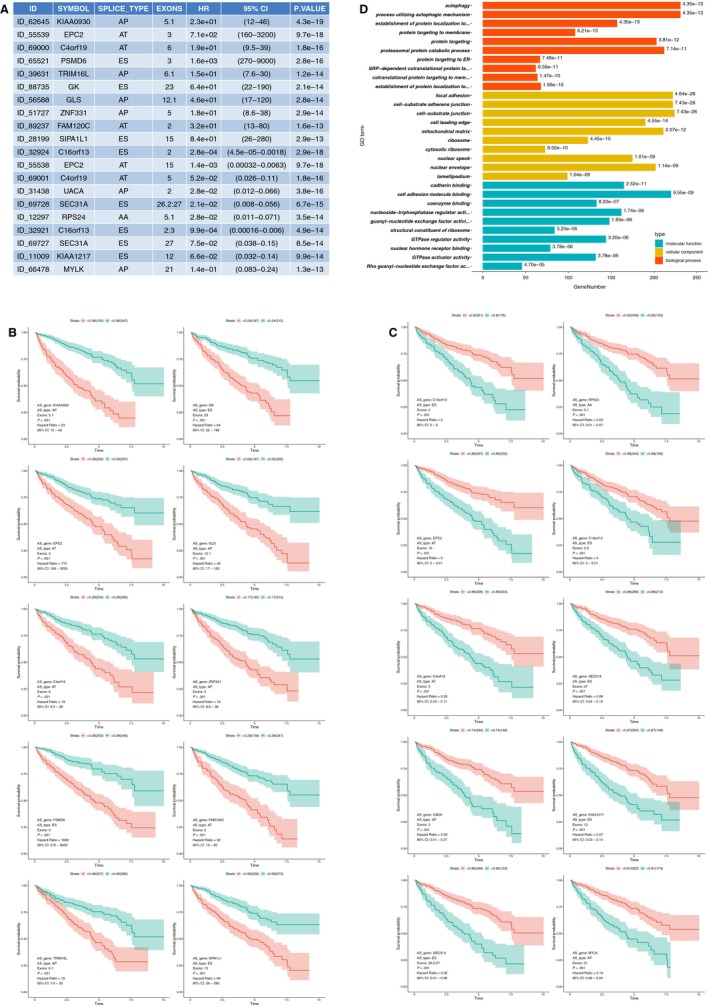
Survival‐associated AS events in KIRC. A, Top 10 hazard ratio (HR) >1 AS events and top 10 HR <1 AS events. B, K‐M curves for top 10 HR >1 survival‐associated AS events. C, K‐M curves for top 10 HR <1 survival‐associated AS events. AS, alternative splicing; CI, confidence interval. D, Top 10 GO terms. Adjust *P* value <0.05 was considered significant. Red columns represent biological process (BP), yellow columns represent cellular function (CF), and blue columns represent molecular function (MF)

To study the prognostic value of mRNA splicing events, we performed univariate regression analysis to identify survival‐associated AS events (both prognostic *P* value and log‐rank *P* < .005 were considered significant). Consequently, we detected 12 888 survival‐associated AS events in KIRC. The top 10 HR >1 and the top 10 HR <1 survival‐associated AS events are presented in Figure 2A, which contains information on gene ID and symbol, splicing type, exon site, HR, 95% CI and *P* value. We discovered that the AT of C4orf19 in exon site 6 was a favourable prognostic predictor, whereas the AT of C4orf9 in exon site 5 was a poor prognostic predictor, indicating that a specific type of splicing event in different exon sites of one gene could have the opposite or distinctly different outcomes in KIRC. The top 10 HRs >1 and the top 10 HRs <1 AS events showed considerable power in distinguishing the good or poor outcome of patients with KIRC (Figure 2.B and C). To speculate the potential functions of survival‐associated AS events, we utilized 6055 source genes of 12 888 AS events for bioinformatics analysis with GO. Top 10 significant GO terms were identified in BPs (adjusted *P* < .05), such as autophagy and their associated pathways, protein targeting and protein catabolic process; furthermore, the top 10 significant MF and CC pathways, such as cell adhesion and their associated pathways, were identified (adjusted *P* < .05) (Figure 2D).

### 3.3 Multivariate AS prognostic model

**Figure 3:**
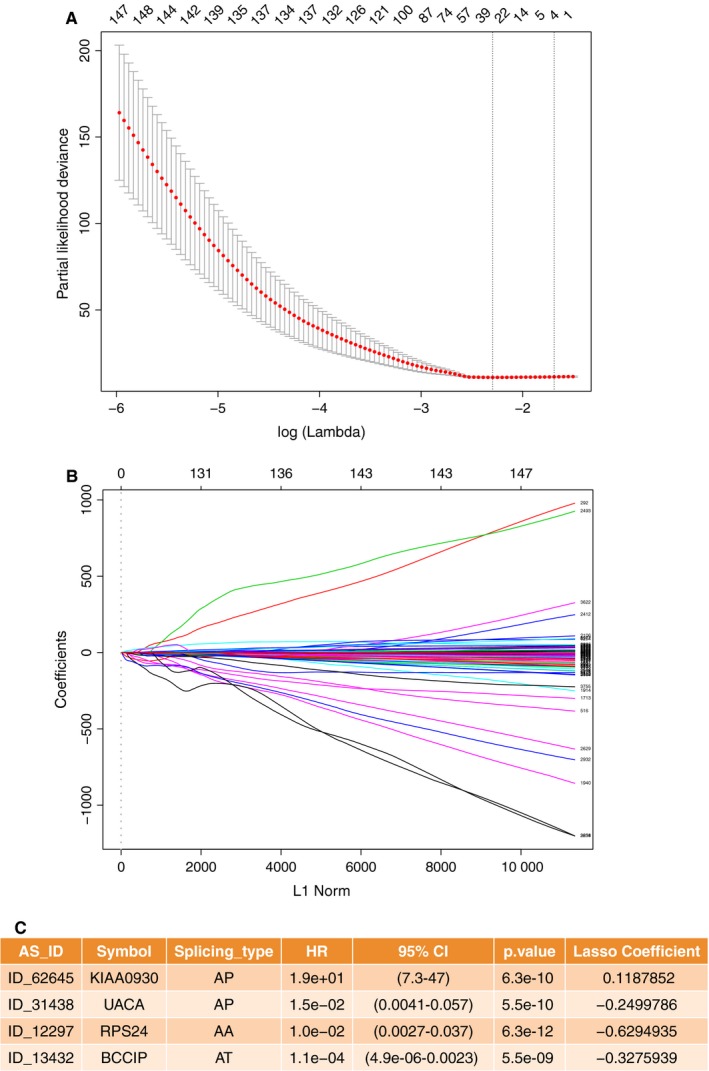
Multivariate prognostic model constructed by LASSO regression. A, Selection of the tuning parameter (λ) in the LASSO model through 10‐fold cross‐validation procedure was plotted as a function of log(λ). The y‐axis represents partial likelihood deviance, and the lower x‐axis represents the log(λ). Numbers along the upper x‐axis represent the average number of predictors. Red dots indicate average deviance values for each model with a given λ, where the model provides its best fit to data. B, LASSO coefficient profiles of the 8632 survival‐associated AS events. The black dotted vertical line was the value selected using tenfold cross‐validation in A. C, Final multivariate prognostic model containing four survival‐associated AS events

After conducting univariate regression analysis, we obtained 12 888 survival‐associated AS events (*P* < .05). We then selected 8632 significant survival‐associated AS events (*P* < .01) as candidates in identifying the final prognostic predictors for patients with KIRC. As shown in (Figure 3A and B), LASSO logistic regression was performed to the 8632 candidate AS events. Certain coefficients were accurately reduced to zero by forcing the total absolute value of the regression coefficients to be less than the constant value, and the most powerful prognostic predictors were selected. The final prognostic model of LASSO regression analysis is presented in Figure 3C as follows: first, the AP of KIAA0930 (HR = 1.9e + 01, 95% CI 7.3‐47, *P* = 6.3e‐10, LASSO coefficient was 0.1187852); second, the AP of uveal autoantigen with coiled‐coil domains and ankyrin repeats(UACA, HR = 1.5e‐02, 95% CI 4.1e‐03‐5.7e‐02, *P* = 5.5e‐10, LASSO coefficient was −0.2499786); third, the AA of ribosomal protein S24 (RPS24, HR = 1.0e‐02, 95% CI 2.7e‐03‐3.7e‐02, *P* = 6.3e‐12, LASSO coefficient was −0.6294935); and fourth, the AT of BRCA2 and CDKN1A interacting protein (BCCIP, HR = 1.1e‐04, 95% CI 4.9e‐06‐2.3e‐03, *P* = 5.5e‐09, LASSO coefficient was −0.3275939). Most of these events were the same as the top important AS events presented in Figure 2A containing the AP of KI990, the AP of UACA and the AA of RPS24. This coincidence of overlapping indicated the science and efficiency of LASSO regression.

### 3.4 Training for the multivariate AS prognostic model

**Figure 4:**
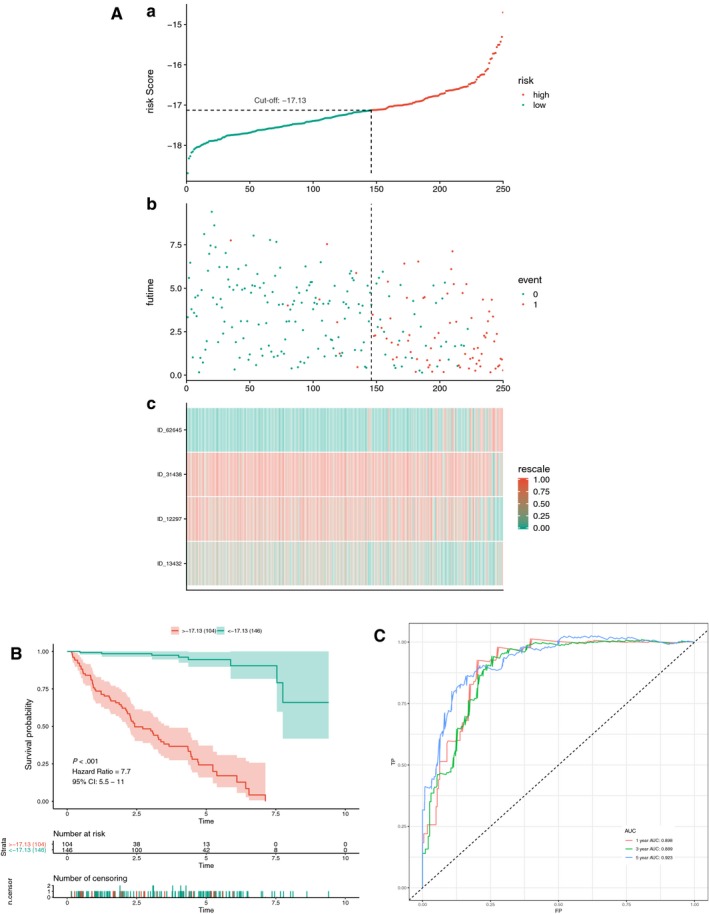
Training for AS multivariate prognostic model. A, Upper part shows the distribution of risk score; the middle shows patients' survival time and status. The black dotted line represents the optimum cut‐off point dividing patients into low‐ and high‐risk groups; the bottom shows the heat map of the PSI value of the final AS predictors. B, Upper part shows the Kaplan‐Meier (K‐M) curves for high‐ and low‐risk groups; the middle shows the number of living patient variation with time in high‐ and low‐risk groups; the bottom shows the number of censoring variation with time in high‐ and low‐risk groups. Green colour represents low‐risk group data, whereas red colour represents high‐risk group data. C, Receiver operator characteristic (ROC) curves for patients under training set at 1, 3 and 5 years. Red colour represents 1 year, green colour represents 3 years, and blue colour represents 5 years. AUC, area under the curve

Initially, a risk score was calculated for each patient in the training set by combining the PSI value of AS and the corresponding LASSO coefficient. The cut‐off point generated from the optimal sensitivity and specificity based on the ROC curve was then used to divide the patients into high‐ or low‐risk groups (Figure 4A and C). Patients with risk scores of ≥ −17.13 were allocated to the high‐risk group, whereas the remaining patients were in the low‐risk group (Figure 4A). Patients with high‐risk scores likely expressed a high PSI value of the risky AS events (HR >1), whereas patients with low‐risk scores tended to express a high PSI value of protective AS events (HR <1) (Figure 4A). To study the relationship between risk score and survival status, we performed K‐M curves and log‐rank test on the training sets. As shown in Figure 4B, patients with high‐risk scores likely had a low survival probability, whereas those with low‐risk scores had a high survival probability (HR = 7.7, 95% CI: 5.5‐11; *P* < .001). As shown in Figure 4C, we used AUCs at 1, 3 and 5 years to assess the prognostic power of the final model. The AUCs of the ROC curves at 1, 3 and 5 years were >0.88, revealing a powerful prognostic ability.

### 3.5 Testing for the multivariate AS prognostic model

**Figure 5:**
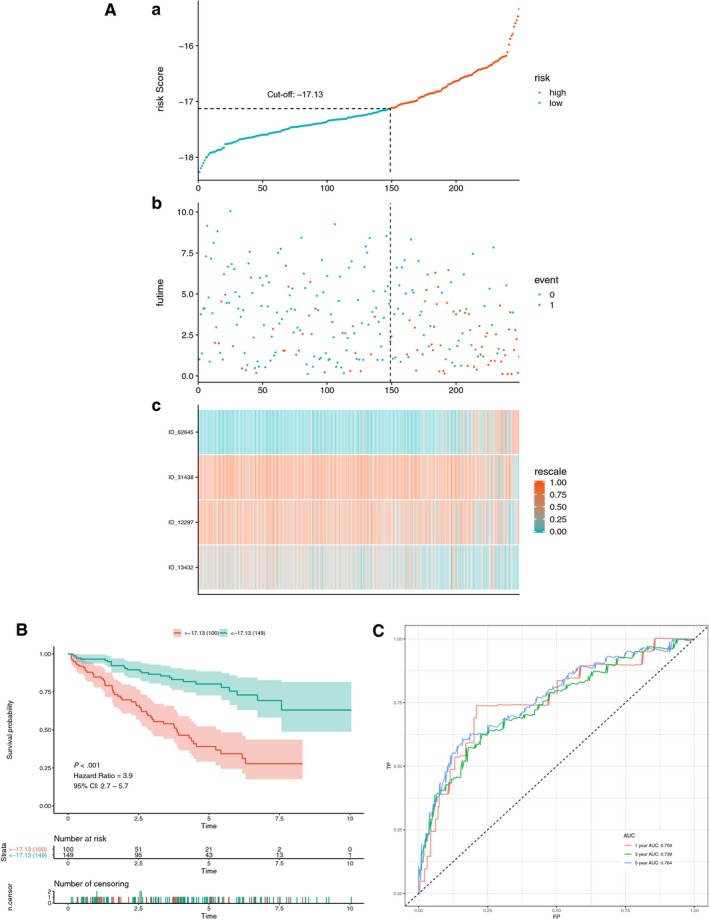
Testing for AS multivariate prognostic model. A, Distribution of risk score and heat map of the genes from the final prognostic model. The black dotted line represents the optimum cut‐off point dividing patients into low‐ and high‐risk groups. B, Survival analysis of patients under the test set. Green colour represents low‐risk group, whereas red colour represents high‐risk group. C, ROC curves for patients under the test set at 1, 3 and 5 years. Red colour represents 1 year, green colour represents 3 years, and blue colour represents 5 years. AUC, area under the curve

To verify the results of the training set, we performed the same analyses on the patients in the test set. As shown in Figure 5A,B,C, the results corresponded to our observation in the training set (HR = 3.9, 95% CI: 2.7‐5.7; *P* < .001). The AUCs in the test set were 0.759, 0.739 and 0.764 at 1, 3 and 5 years, respectively. This observation verified the prognostic power of the final prognostic model in patients in the test set (Figure 5C). Hence, the prognostic model could satisfactorily predict the prognosis of patients with KIRC.

### 3.6 Potential functions of genes from AS predictors in the multivariate AS prognostic model

**Figure 6:**
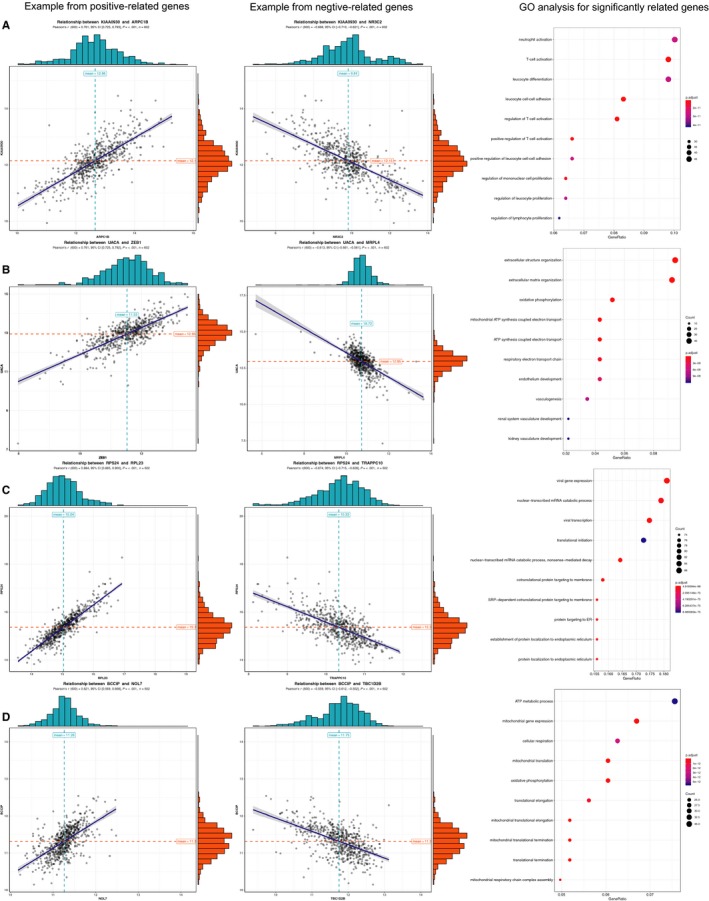
Correlation analysis and GO analysis for the source genes of AS in the multivariate prognostic model. A, Positive correlation between the expression of KIAA0930 and ARPC1B, and negative correlation between the expression of KIAA0930 and NR3C2. GO in BP for top 500 protein‐code genes correlated with KIAA0930. B, Positive correlation between UACA and ZEB1, and negative correlation between UACA and MRPL4. GO in BP for top 500 protein‐code genes correlated with UACA. C, Positive correlation between RPS24 and RPL23, and negative correlation between RPS24 and TRAPPC10. GO in BP for top 500 protein‐code genes correlated with RPS24. D, Positive correlation between BCCIP and NOL7, and negative correlation between BCCIP and TBC1D2B. GO in BP for top 500 protein‐code genes correlated with BCCIP. Person correlation analysis was performed, and all the absolute values of regression coefficient >0.5 and *P* < .05. Adjust *P* value in GO are all <.05

We analysed the co‐expression between genes from the final AS predictors and other protein‐code genes to obtain a co‐expression relationship. (Figure 6A–D) illustrates the positive and negative correlations as examples. We then chose the top 500 correlated protein‐code genes based on regression coefficients and *P* < .05 to perform GO and speculate the functions of genes from the final AS predictors. The top significant BPs of KIAA0930 correlative genes were mainly associated with immunocyte and inflammatory cell pathways, including T cell, neutrophil, leucocyte, mononuclear and lymphocyte (Figure 6A). The significant GO terms of UACA correlative genes indicated that extracellular structure and oxidative phosphorylation were associated with ATP pathways (Figure 6B). The significant GO terms of RPS24‐related genes indicated viral gene expression, including transcription and translation (Figure 6C). The significant GO terms of BCCIP‐related genes corresponded to pathways related to ATP metabolism (Figure 6D). Most of the abovementioned GO terms were closely associated with oncogenesis, and this finding revealed the potential functions of the final prognostic model influencing the outcome of patients.

## 4 DISCUSSION

In the present study, we first demonstrated the comprehensive feature of seven different types of AS in KIRC and then provided important information about the systematic analyses of AS events in KIRC. Consistent with other studies, our research showed that AS is a common process in KIRC and involved in oncogenesis. We identified massive AS events closely associated with survival via univariate regression analysis. We uncovered the potential functions of these AS events through GO analysis. Among BP pathways, autophagy is the most remarkable and widely explored as a vital factor of oncogenesis, tumour progression and cancer therapy.^35^, ^36^, ^37^ Moreover, autophagy plays an important role in KIRC.^38^, ^39^ Protein targeting and catabolic process are associated with oncogenesis.^40^ Aberrant cell adhesion is normally associated with tumour progression and cancer metastasis.^41^, ^42^ Our results indicated several top GO terms closely related to cell adhesion in CC and MF. The top significant GO terms were closely connected to oncogenesis, demonstrating that the results of GO analysis were reasonable. In addition, combining the results of GO analysis and previous studies suggested that survival‐associated AS events potentially influenced the abovementioned pathophysiological processes leading to KIRC.

We systematically studied the prognostic value of AS events in patients with KIRC. By applying LASSO regression analysis for survival‐associated AS events, we first constructed an independent and efficient prognostic model, including AP of KIAA0930, AP of UACA, AA of RPS24 and AT of BCCIP. Traditionally, multivariate COX regression is chosen to build a multivariate model, which generally focuses on several variables.^19^, ^20^, ^43^ By contrast, LASSO regression is preferably suitable for the regression of massive and multivariate variables. To verify the prognostic model, we first constructed a novel classifier consisting of four AS events in the training and test sets. The results of K‐M curves in the training and test sets revealed that the final prognostic model could successfully subdivide patients into a high‐ or low‐risk group with poor or favourable OS, respectively. In comparison with other studies, this study showed that the results of AUCs of ROC curves manifested favourable sensitivity and specificity. For instance, the AUC in the prognostic model of prostate carcinoma is 0.756, and the AUC in bladder carcinoma is 0.748, indicating acceptable sensitivity and specificity.^21^, ^43^ Thus, by calculating the risk scores combining the PSI value of AS and the corresponding LASSO coefficient, we constructed a powerful high‐performance prognostic model for risk stratification in KIRC, and it had promising implication in clinical practice. Given the high prevalence of splicing defects in cancer, the small‐molecule modulators of RNA processing showed potential for novel therapeutic strategies in cancer treatment. Therefore, the AS events in this model could be targets in KIRC therapy.

Genes with correlative expression levels perform similar functions.^44^ Though the roles of the final AS predictors and their source genes in KIRC have not yet been studied, we performed co‐expression and GO analyses to speculate the function of these AS source genes. For the four AS events in the prediction model, the most significant BPs were closely associated with inflammatory cell activation, oxidative phosphorylation and ATP metabolism‐associated pathways, which are potentially related to cancer cell activation and changes in cellular metabolism and immunological function in KIRC.^45^, ^46^, ^47^, ^48^ Thus, the prognostic model could differentiate the prognosis of patients with KIRC and provide scientific evidence for future in‐depth studies on AS mechanisms in KIRC.

However, this study has limitations. For example, our results were verified only by the TCGA data set because an additional external data set is unavailable. In addition, the prognostic model is not yet clinically validated. Thus, our next work may focus on clinical evaluation and application after prospective clinical trials. In conclusion, we unveiled the systematic feature of AS events in KIRC for the first time and highlighted this field. Second, we built a powerful prognostic model combining multiple types of AS events in KIRC and possibly provided a significant basis for conducting future clinical studies. This study discovered massive important AS sites and presented scientific data for further mechanism studies.

## 5 FUNDING STATEMENT

This work was supported by the NSFC (Grant no. 81270805) and the Science and Technology Department of Sichuan Province (Grant no. 2018SZ0378).

## CONFLICT OF INTEREST

On behalf of all authors, the corresponding author states that there is no conflict of interest.

## AUTHOR CONTRIBUTIONS

WT designed the experiments; WT and WH.T. analysed the data; and YZ and LZ wrote the manuscript. All authors read and approved the final manuscript.

## Data Availability

All data sets generated/analysed for this study are included in the manuscript and the [Link], [Link], [Link].
